# A Genetic Algorithm Procedure for the Automatic Updating of FEM Based on Ambient Vibration Tests

**DOI:** 10.3390/s20113315

**Published:** 2020-06-10

**Authors:** Francesca Bianconi, Georgios Panagiotis Salachoris, Francesco Clementi, Stefano Lenci

**Affiliations:** Department of Civil and Building Engineering, and Architecture, Polytechnic University of Marche, Via Brecce Bianche n.12, 60131 Ancona, Italy; f.bianconi@pm.univpm.it (F.B.); g.p.salachoris@pm.univpm.it (G.P.S.); francesco.clementi@univpm.it (F.C.)

**Keywords:** monitoring, masonry structures, operation modal analysis, dynamic identification, model updating, calibration

## Abstract

The dynamic identification of the modal parameters of a structure, in order to gain control of its functionality under operating conditions, is currently under discussion from a scientific and technical point of views. The experimental observations obtained through structural health monitoring (SHM) are a useful calibration reference of numerical models (NMs). In this paper, the procedures for the identification of modal parameters in historical bell towers using a stochastic subspace identification (SSI) algorithm are presented. Then, NMs are manually calibrated on the identification’s results. Finally, the applicability of a genetic algorithm for the automatic calibration of the elastic parameters is considered with the aim of searching for the properties of the autochthonous material, in order to reduce modelling error following the model assurance criterion (MAC). In this regard, several material values on the same model are examined to see how to approach the evolution and the distribution of these features, comparing the characterization proposed by the genetic algorithm with the results considered by the manual iterative procedure.

## 1. Introduction

Recent technological and code developments within civil engineering require buildings to be designed and verified from both a static and dynamic point of view. Studying the dynamic behavior of a structure implies understanding how it reacts when it is subjected to external dynamic forces, such as earthquakes or even just a general environmental action.

In many parts of Europe, and in particular in Italy, the main concern is how to protect structures from seismic events. Italy is a country characterized by a high seismic risk, due to its own features: frequency and intensity of earthquakes, high population density and, above all, a vulnerable building heritage, in particular historical ones like churches [1,2,3] and clock towers [4,5]. Historical centers are usually composed by masonry structures designed and built according to obsolete construction codes or, for what concerns the most ancient sites, with no regulations but the experience of masters. The age of buildings and materials, along with the lack of appropriate maintenance, negatively affect the global performance of existing constructions. All these specific problems lead to a search for accurate numerical models (NMs), usually with the finite element method (FEM) [6,7,8,9], through which it is possible to understand the real conditions of the structure with the intention of making predictions on its dynamic behavior [10] and then design and plan effective maintenance interventions. To create these NMs, the information that can be collected are generally extremely poor and unreliable, so assumptions are often made about the real materials used, their state of conservation and their texture, the type of connections between elements, the presence of tangible damage, even if these are the features that most influence the behavior of the structure [11,12]. In this context it is essential to control the functionality of the structure under operating conditions. To achieve this goal, the structural health monitoring (SHM) approach is used, through which experimental observations fundamental for the diagnostic process of a building are made [13,14,15] and its results may be used to calibrate a NM and make it more similar to real behavior.

The SHM process involves the observation of a system (e.g., bridge, tower, church, etc.) over time using periodically sampled dynamic response measurements from an array of sensors, the extraction of damage-sensitive features from these measurements, and the statistical analysis of these characteristics to determine the current state of system health [16,17]. For long-term SHM, the output of this process is periodically updated, giving information on the ability of the structure to perform its intended function along time, considering the inevitable aging and degradation resulting from operational environments [18,19]. After extreme events, such as earthquakes, or accidents such as blast loading and floods, SHM is used for a rapid condition screening to provide, in near real time, reliable information regarding the integrity of the structure [20,21,22]. Frequently, the calibration of NMs is also useful in particular when the localization and quantification of damage are the main issues to explore, obtaining a perfect match in terms of frequencies and modal shapes.

In general, when the observation time is short, typically when only an identification of the dynamic characteristics is the priority, two different techniques are available depending on known prior input. The first case is the experimental modal analysis (EMA) [23,24] for which the dynamic behavior of the structure is investigated after the application of a known exciting force. This methodology is rarely used because it is more demanding (and more expensive). The operation modal analysis (OMA) instead is a simpler non-invasive dynamic identification technique (e.g., it does not require to stop the operativity of the building), which allows to evaluate the modal parameters when the structure is excited by an unknown force, such as traffic, wind, etc. [25], and only the output is measured.

In this paper, the OMA technique is used in short-time tests on a masonry bell tower. The monitoring was performed in one day making various acquisitions at different positions of the accelerometers. The obtained parameters were then used for a manual calibration of the NM modifying the elastic properties of the materials assigned in the model [13,26] given the availability of a high precision geometric survey. Subsequently the same calibration was automatically performed through a genetic algorithm [27,28]. Finally, the differences obtained from the two procedures are analyzed [29,30,31,32].

## 2. The Case Study

**Figure 1:**
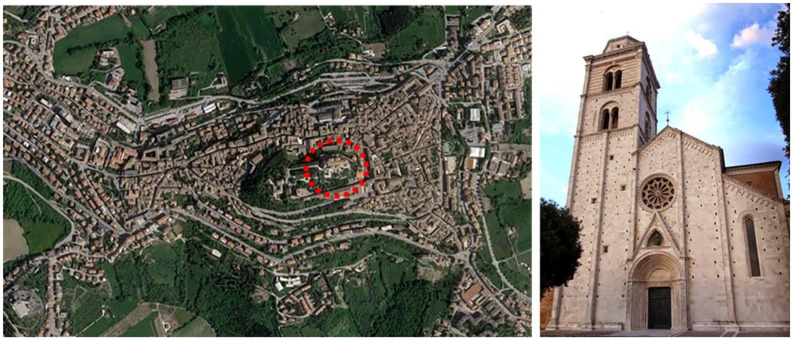
Metropolitan Cathedral of Fermo location (Marche, Central Italy).

The subject of the study is the bell tower of the Metropolitan Cathedral of Fermo (Marche Region, Italy), dedicated to Santa Maria Assunta in Cielo (Figure 1). To reach the current layout, the cathedral has undergone several changes over the centuries. The first traces of its existence date back to the early Christian age, probably the 2nd century AD. In 1176 the existing church was destroyed by a fire caused by the siege of the city by the troops of Frederick I (also known as Frederick Barbarossa), and in 1227 the new cathedral was built in Romanesque-Gothic style on the initiative of Bartolomeo Mansionario, based on a project by the architect Giorgio da Como. At the behest of Archbishop Andrea Minnucci, the church was demolished and rebuilt between 1781 and 1789, based on a design by the architect Cosimo Morelli, but the facade and the belfry were preserved. The characteristic asymmetry of the facade is due to the construction of the bell tower in correspondence with the church aisle. Thus, the top of the facade does not correspond to the position of the portal and the rose window. The last significant renovation of the bell tower took place in 1898.

The cathedral was damaged by the seismic sequence of Central Italy of August and October 2016 [33] and has been closed for restoration for more than one year.

**Figure 2:**
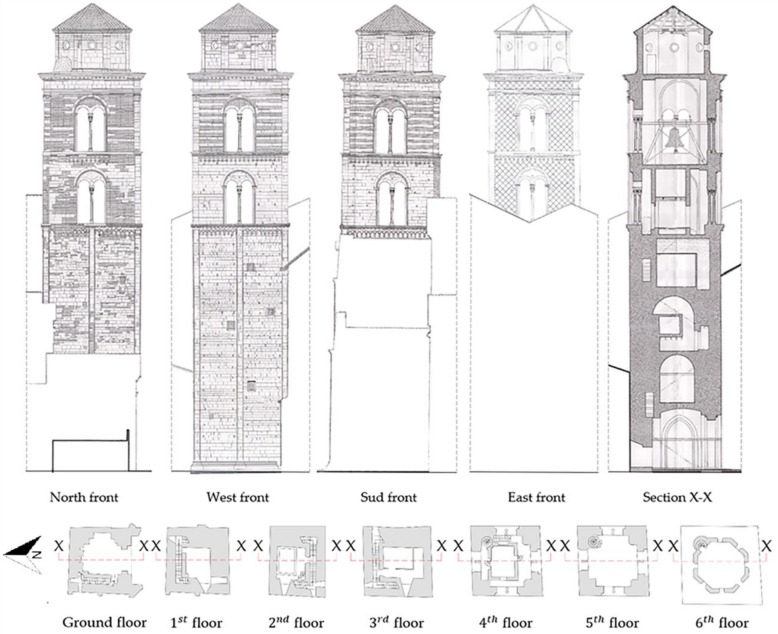
Geometrical features of the bell tower.

The bell tower is linked on the South side with the facade and on the East side with the church. It is 48.1 m tall and presents a square section plan (10 × 9.5 m). The walls, for a height of 24.3 m, are 2.7 m thick and a stairwell is located inside them. The fourth and fifth floor have walls 1.5 m thick and there is a window on each side, consisting of two arched openings resting on two columns. On the fifth level, five bells are installed on a complex steel structure. The top floor is octagon shaped. On each side, approximately 3.1 m long, there is a circular window and the walls are 0.5 m thick. These last three levels are connected by a spiral staircase located on the northeast corner (Figure 2).

Regarding the belfry’s constructive typology, from the information collected and the inspection conducted, the walls are constituted by solid bricks and stone (Istrian stone), the floors are made of masonry vaults and the cover floors consists of wooden trusses.

## 3. Structural Health Monitoring

As mentioned above, SHM provides the real dynamic behavior of a structure. This aspect is fundamental since NMs often return dynamic properties that differ from the real ones for several reasons, such as: geometric approximations, assumptions regarding the parameters of the materials that are assigned, the connections between elements or non-visible internal damage states. In order to overcome these issues, using the OMA results it is possible to update the NM to make it closer to reality, monitoring the vibrations of a structure produced by an unknow input under operating conditions. A monitoring system consists of a set of sensors located at strategic points of the structure [34] that communicate with a central acquisition unit. The acquired data will be processed and used to create the experimental model (EM) used to calibrate the NM.

### 3.1. Data Acquisition and EM Development

Three triaxial GEA II sensors (Sequoia, Moncalieri, Italy) were used. These are hybrid piezo-MEMS sensors with maximum measurable value of 8 g, sensitivity of 1 V/g, high dynamic range of 120 dB due to the 24-bit A/D converter. These sensors are connected using cables up to 100 m long to a sync Hub (Sequoia, Moncalieri, Italy), which allows them to be synchronized, connected to the acquisition unit. The data were acquired and recorded by using the GEA Lab© software (Sequoia, Moncalieri, Italy).

**Figure 3:**
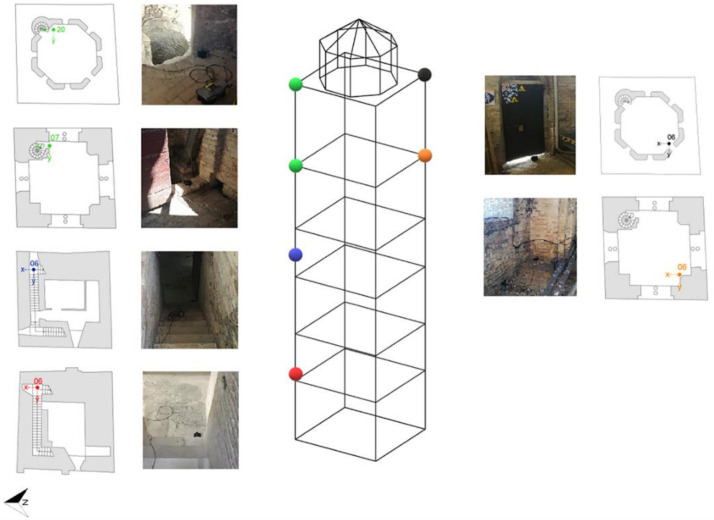
Sensor layout.

Four measurements were obtained (Figure 3): two sensors were left fixed in the highest part of the tower (green color), while the position of the third one was modified at each acquisition, to obtain information about the dynamic behavior of a significant part of the tower. The sensors were fixed through a resin, avoiding any possible damage to the structure, to make sure they were correctly connected to the structure.

To exclude the effects of possible non-stochastic vibration that may occur during the measurements, it is necessary to consider at least 2000 times the highest expected period of the structure in order to establish the duration of recordings [35]. To satisfy this empirical formulation, the acquisitions last 45 min. The instrumentation allowed sampling at 1024 Hz.

The acquisition program offered us the chance to represent the EM under the structure of nodes and lines. The related nodes were attached to the information regarding the corresponding sensors and the registered signals in terms of vectors and reference positions. In this way it was possible to represent the geometry of the structure in tandem with the eigenvalues and eigenvectors of the Stochastic Subspace Identification (SSI) analysis [36]. The wireframe of the model consists of 749 nodes to grant a better understanding of the mode shapes displacement under different interpolations. This may be regarded as a surrogate model, treated as a base for calculations to optimize problems of eigenvectors and eigenvalues. Before analyzing the raw data, a decimation of the frequencies 0–12.8 Hz was set, consistent with the real frequencies of a structure of this type.

### 3.2. Dynamic Identification

The identification of the frequencies was implemented using the (SSI) method [37], developed in the time domain. To obtain the modal parameters, a discrete-time state-space representation is used:

(1) $${\hat{x}}_{k+1}=\left[A\right]\cdot {\hat{x}}_{k}+\left[B\right]\cdot u_{k}$$

(2) $$y_{k}=\left[C\right]\cdot {\hat{x}}_{k}+\left[D\right]\cdot u_{k}$$

where $x_{k}=x_{k}\left(\Delta t\right)$ is the discrete-time state vector containing displacements and velocities of the system, $y_{k}$ is the vector of system response, $u_{k}$ is the vector of input, [*A*] and [*B*] are the matrices of input that contain, respectively, the physical information and the statistical parameters, [*C*] is the output matrix and [*D*] is the direct transmission matrix in discrete time.

The main concern of this parametric model estimation is that the true order of the model is unknown, due to the noise content that may be present in the recorded signals or limited recordings. The strategy is to consider several models with an increasing order to identify all physical modes in the frequency range of interest. However, this also involves the presence of many mathematical modes (spurious modes) that have no physical meaning and are simply due to the noise content of the recordings. 

**Figure 4:**
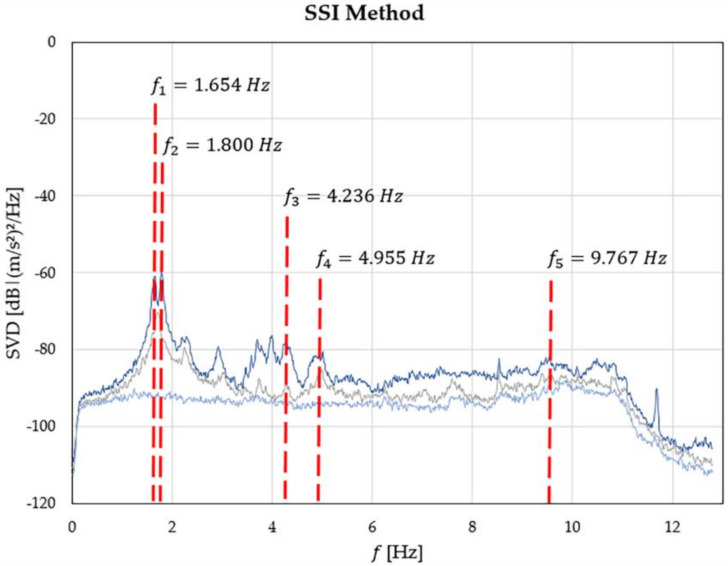
Frequency estimation graph.

A stabilization diagram (Figure 4) is used to separate the physical and spurious modes. Through this tool it is possible to see graphically the natural frequency of the system as the alignment of points (stable modes) given by the various models considered. Five main frequency were identified. Due to the number of sensors used and their position within the structure, between the second ($f_{2}$ = 1.800 Hz) and the third ($f_{3}$ = 4.236 Hz) frequencies no other stable modes could be individuated.

**Figure 5:**
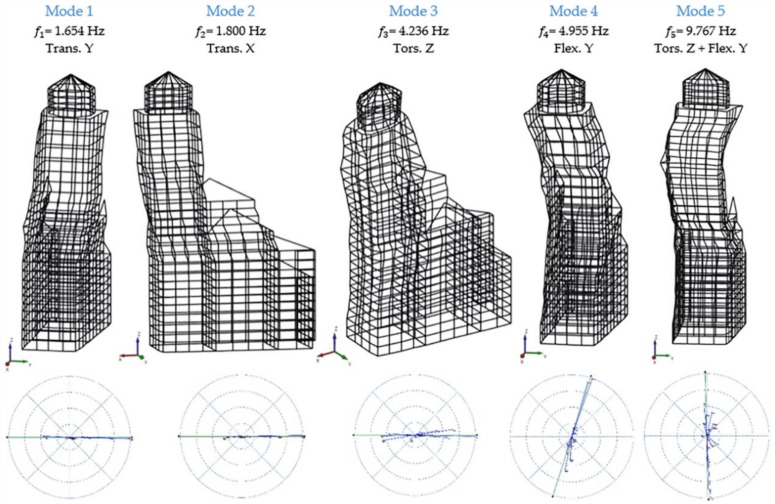
Experimental model: mode shapes, frequencies and complexity plot.

Figure 5 shows the modal shapes of the bell tower, to which the frequencies of the stabilization diagram correspond. It appears that the first two modes are translational, the third seems to be rotational, the fourth is flexural and fifth is flexural with a torsional component. To know the reliability of these modes, a useful tool is the *Complexity Plot*. One mode is reliable if it is not complex and to achieve it, the shape components should form along a straight line (Figure 5). With a more careful examination, the third mode also has a distortion component of the transversal section. There are no references to this mechanism in the literature and it may be eventually be the topic of future investigations.

## 4. FE Modeling

The development of the NM, using the MidasFea© software (Midas, Seongnam-si, Korea) started with the geometric representation. In this phase it is important to pay attention to the elements that affect the dynamic behavior of the structure (such as masonry vaults, openings, walls’ thickness, wooden roof, niches, etc.), while unnecessary secondary elements can be neglected and considered only as masses.

**Figure 6:**
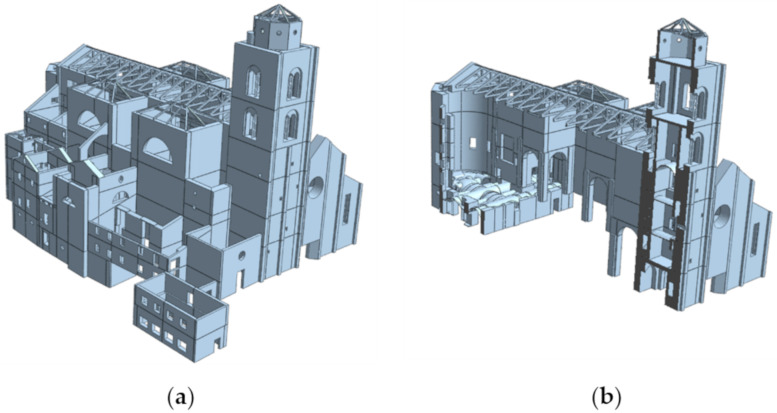
Numerical Model: (**a**) prospective view; (**b**) section view.

Figure 6 shows the NM in its totality. We decided to build each part of the structure because the real interaction between the bell tower and the church was unknown. The bell tower was built with masonry brick and lime mortar and Istrian stone. The materials’ properties, chosen according to the Italian Technical Standards for Structures [38] considering the lowest limit, were then assigned to solids which are discretized as 4-nodes tetrahedral solid elements. The elastic parameters assigned in the model were also corrected with coefficients reported in Table.C8.5.II of [39] because of some limitations such as a mere visual inspection and the lack of information of the material used and their conservation status.

Finally, the constraints at the base were assigned as fixed and the loads given by the floors and the bells were included in the model. In the starting model (step 0) the Young’s Modulus (E) was considered uniform and equal to 1800 MPa.

**Figure 7:**
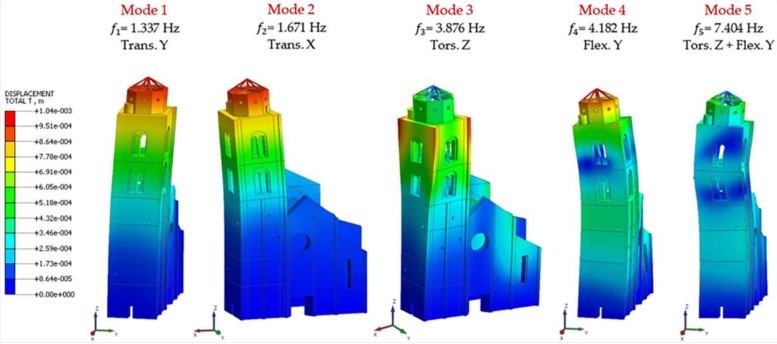
Numerical Model: mode shapes and frequencies at step 0.

Since the first modes deriving from the modal analysis basically involved the tower, the principal modes of the belfry were investigated. In order to maximize the visualization of the modal shapes of the belfry, the next figures exclude the church main construction, considering it not relevant for the results of this paper, as will be shown thereafter. The main numerical modal shapes are reported in Figure 7, and from a quick check, they appear quite responsive with the EM’s modal shapes. Instead, Table 1 compares the experimental and numerical frequencies and it shows that the difference is quite large.

### 4.1. Model Updating

To match the results, an update of the NM is required. The calibration was obtained with an iterative manual procedure in which the Young’s Modulus E (MPa) of the assigned materials is updated. The aim is to obtain the correspondence of modal shapes and frequencies of the NM with modal shapes and frequencies of the EM.

A visual inspection showed that the Istrian stone is concentrated in the first half of the structure. Thus, a first model was reproduced, dividing the bell tower into sections with decreasing elastic modulus from bottom to top. In total, four different elastic modules were assigned (step 1), significantly increasing all frequencies except the third one (Table 2).

**Figure 8:**
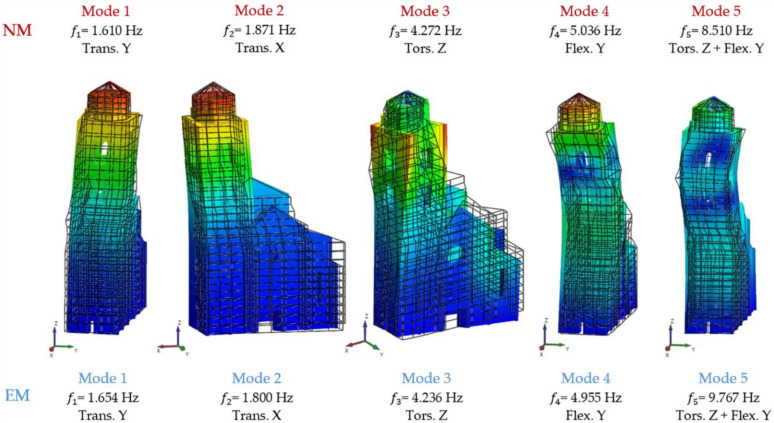
Comparison between the Numerical Model (NM) and Experimental Model (EM) mode shapes at step 2.

The Young’s moduli were again modified along the thickness of the walls, considering that for a multi-leaf masonry walls the presence of the Istrian stone is only on the outside leaf. Along with these improvements, all frequencies are calibrated, except for the second and the fifth ones, assuming an error of 5% as acceptable. In order to reduce the second frequency, considering that the portion of the tower in the north side appears to be made of inhomogeneous material, the elastic modulus in that side was greatly decreased. This last model was obtained by assigning twenty-tree different variations of E to the materials (step 2). Figure 8 and Table 3 compare the modal shapes and frequencies obtained from the NM of step 2 with those of the EM. It was not possible to obtain the correspondence of the fifth frequency, even though the error was considerably reduced.

### 4.2. CrossMAC Matrix

From the reported results, a good (quantitative) match in terms of frequencies was obtained with the manual calibration procedure. A further comparison may be made to have a quantitative measure between EM and NM also for modal shapes by using the modal assurance criterion (MAC) [13], defined as:

(3) $$crossMAC_{ij}=\frac{{\left(\Phi_{i}^{H}\;W{\;\Phi }_{j}\right)}^{2}}{\left(\Phi_{i}^{H}\;W{\;\Phi }_{i}\right)\left(\Phi_{j}^{H}\;W{\;\Phi }_{j}\right)},$$

where $\Phi_{i}$ is the *i*-th vector of the first base, $\Phi_{j}$ the *j*-th vector of the second base and *W* a weighting matrix that can be, for example, the stiffness or mass matrix.

**Figure 9:**
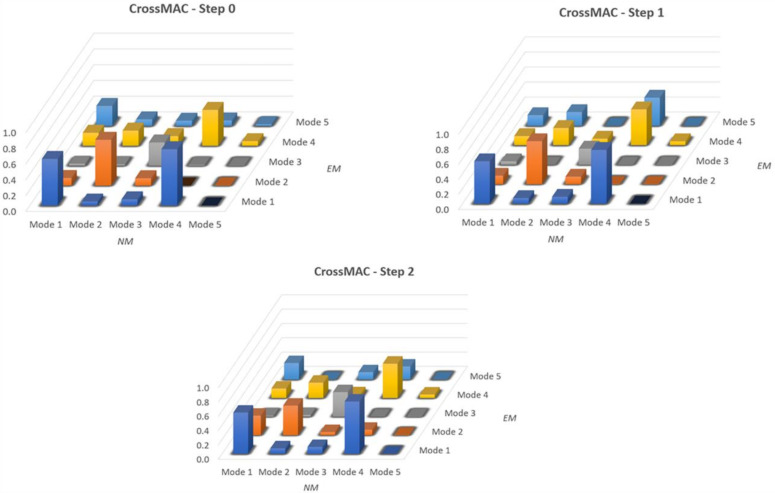
Cross-Modal Assurance Criterion (CrossMAC) between EM and NM.

The optimal result is obtained when the values on the diagonal are equal to 1, while outside the diagonal are around 0. Figure 9 shows the CrossMAC between the EM and NM in all calibration steps. It is noted that CrossMAC does not provide very satisfactory values. In all three cases the highest value occurs for the first mode (respectively: 0.592–0.564–0.574), decreases to second and third mode and then rises to fourth. In the fifth mode the value is always kept very low.

## 5. Model Updating with the Use of a Genetic Algorithm

Following the previously mentioned iterative process, a different procedure for the FEM model updating was implemented. This procedure exploits a genetic algorithm to identify the corresponding eigenvalues and eigenvectors identified by the SSI approach directly on a tridimensional model. The genetic algorithm used is built within in the open source software Code Aster© (Electricité de France (EDF), Paris, France), a free FEM code for numerical simulation of materials and mechanical structures, developed by Electricité de France (EDF). It may be observed in the works [14,29,30,31,40] that different approaches were presented for the study of damage and flaw detection of structures, such as the Latin hypercube sampling and Bayesian model updating that confirm the effectiveness of such algorithms in engineering fields of interest. While the Latin hypercube sampling (LHS) is a statistical method that generates random samples of parameter values coming from a multidimensional distribution, and the Bayesian model updating is a model approach that uses probability to represent all uncertainty within the model, the genetic algorithm [27,28,41,42] is a heuristic search approach that reflects the process of natural selection.

### 5.1. Genetic Algorithm Functionality

The selection of optimal parameters for learning tasks is a challenging step, especially in the context of SHM where the output-only modal parameter identification comes with a high level of uncertainty. The issue becomes increasingly delicate once the identification of the eigen-properties must be done on a Finite Element (FE) model. It is possible to approach such problems in an iterative way, as discussed in the previous chapter, but in order to reduce time and consider a bigger range of possible solutions, it was decided to implement the use of a genetic algorithm.

**Figure 10:**
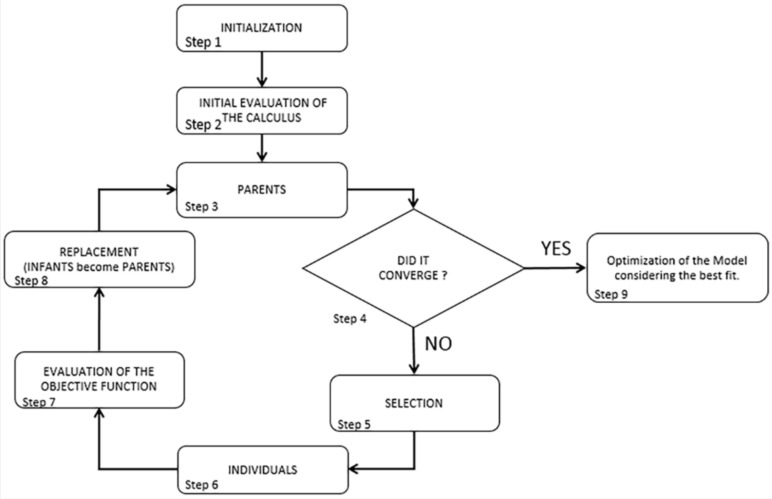
Flowchart representation of the generic algorithm.

The algorithms’ functionality becomes clearer as it is explained in steps (Figure 10). In the first steps (Step 1–3) it generates an initial population from the parameters values that are mapped to the material parameters. These parameters were thought to be constrained by minimum and maximum values. The minimum value was set at 800 MPa and the maximum value at 5500 MPa, as described within the Italian Code [38] for the existing masonry. Then a first analysis is run to create the parental units that become the first measure of comparison (Steps 3–4). If no convergence is achieved, new possible solutions (individuals, Steps 5–6) are generated and the problem evolves itself towards even better solutions (Steps 7–8).

Each candidate has its own set of properties that can be altered. Generally, solutions are represented as binaries of strings with values of 0 s and 1s. The evolutionary process starts from the initial population where all processes start together and, in each iteration (generation), the fitting of every individual process is evaluated. This adjustment is usually the objective function solved in the optimization problem. The better and more adequate individuals are selected in a stochastic manner from the initial population and all their features are furtherly altered and combined to form a new, stronger generation. The new generation of possible solutions (also called individuals) is then used for the next iteration of the algorithm.

The algorithms functionality comes to an end (Step 9) when the maximum iterations is achieved or when the best value of the functional calculus is presented. The results from the optimization came to be the calibrated frequency values and the MAC between the EM and NM.

### 5.2. Model Calibration and Results

**Figure 11:**
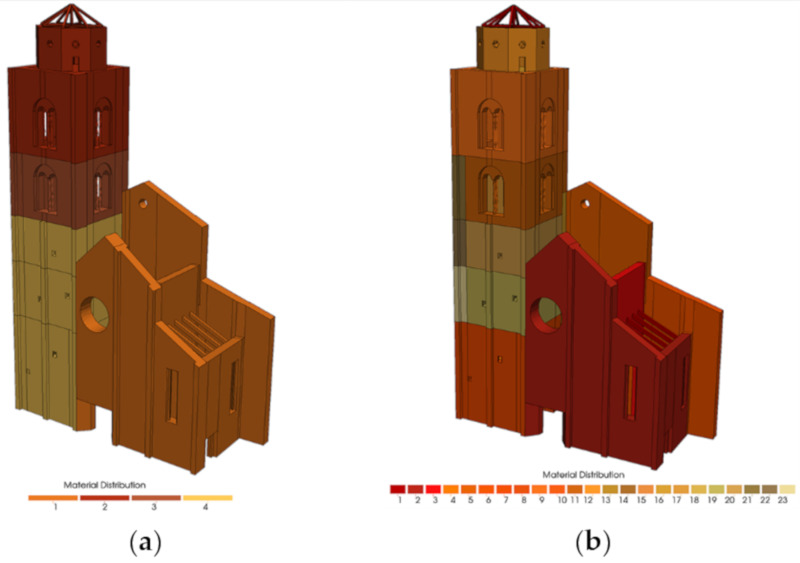
Colormap of the NM with (**a**) four different material values distributed and with (**b**) twenty-tree material values distributed.

As for the manual procedure (Section 4), two types of models are now considered (Figure 11): one with four material values (step 1 of manual update) and one with twenty-three material values (step 2 of manual update) to mark any difference from the genetic algorithm with Code Aster© (Electricité de France (EDF), Paris, France). The materials’ properties were always assigned to tetrahedral solids elements.

Firstly, the modal analysis results of the NM of step 0 with Code Aster© are checked and compared with those of Midas FEA© (Midas, Seongnam-si, Korea) (Figure 7 and Table 1) obtaining a perfect match in terms of frequencies and modal shapes. For this reason, they will not be reported.

**Figure 12:**
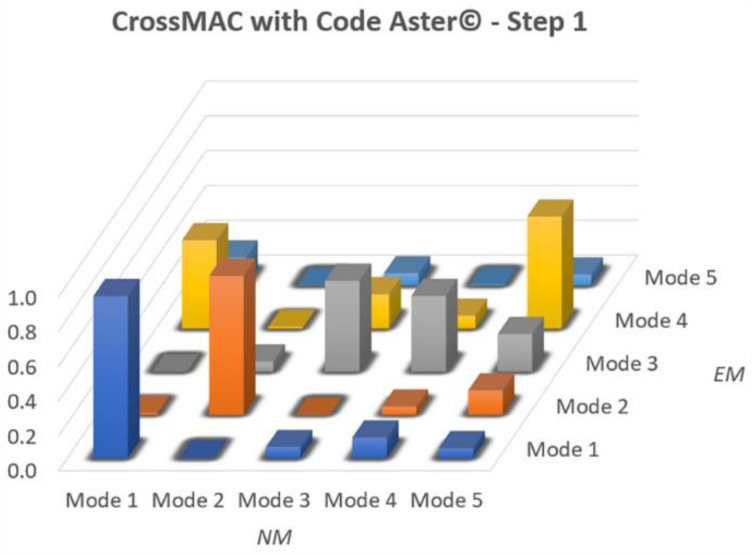
CrossMAC between EM and NM at step 1 with Code Aster©.

The NM of step 1 of the manual update, i.e., with four values of Young’s modulus, was therefore implemented in Code Aster© for the automatic MAC optimization through the genetic algorithm. The main results after the optimization process are reported in Table 4 and compared with those of the EM, and Figure 12 shows the optimized MAC. With the calibration of the Young’s Modulus a better fitting in terms of frequencies are visible: for the main one the error respect to the EM goes from 9.31% of the manual to 0.62% of the actual. The other numerical frequencies remain stationary with respect to manual calibration maintaining a high discrepancy with the EM.

Checking the MAC reported in Figure 12, there is a slight improvement, especially for the main frequencies (~0.8), always far from the unit value. Furthermore, a decrease in the terms of the main diagonal for the fourth and fifth modes was remarked. It is also noted that outside the main diagonal, the noise reported in the manual update of step 1 diminished considerably, even though it is still present. Finally, there is a clear difference regarding the values of Young’s Modulus between the manual calibration and the automatic one (Table 5). This difference is significant, since the algorithm will then try to find the values that better achieve convergence in five different sets.

**Figure 13:**
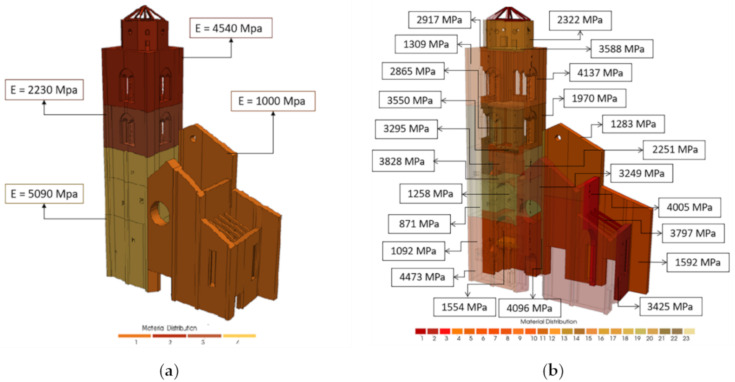
Assigned values of the Young’s Modulus produced by automatic calibration for the NM of step 1 (**a**) of step 2 (**b**).

In Figure 13a it may be observed that the values of the Young’s modulus of the belfry is reduced where the interaction with the facade is predominant. The value goes through 5090 MPa to 2230 MPa and this means possible damage due to pounding, and again to 4540 MPa. The facade of the belfry was considered less important by the algorithm and it was assigned a lower value of Young’s Modulus (1000 MPa) in contrast to the other three elastic parameters.

The genetic algorithm procedure was finally applied to the model with twenty-tree material parameters (Step 2 of the manual calibration). Table 6 shows the results obtained in terms of frequencies. It is clear that the third mode resulted in the biggest deviation between the experimental frequency values, even with the calibration carried out with an elevated number of parameters.

**Figure 14:**
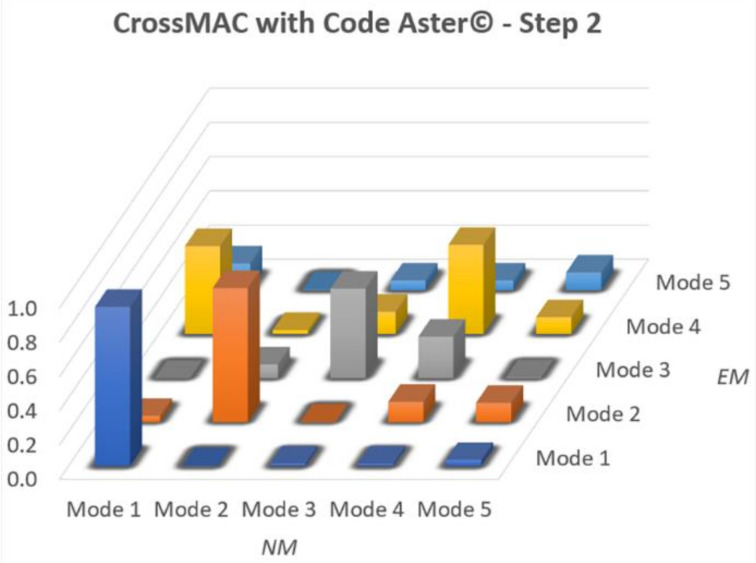
CrossMAC between EM and NM at step 2 with Code Aster©.

Although the numbers of the optimized frequencies of the NM of step 2 are not in complete accordance with the experimental data, the MAC (Figure 14) resulted higher in the diagonal row especially between the third (0.52) and fourth (0.5) modes. The one thing that was also seen in the initial phase, is the spike in the value of the Mode 4 (0.51) which continues to be even at the end of the optimization of the NM of step 2. This value could be mainly due to a lack of information from the experimental data caused by a not optimal position of the accelerometers to catch this mode shape. Compared to the manual procedure, besides having better values on the diagonal, on this step 2 NM the dispersion outside the diagonal is much reduced. It can also be seen that in both procedures the fifth mode is difficult to define and maintains a very low value.

Finally, in Table 7 the values of the twenty-three Young’s moduli after automatic calibration are reported and compared with the same of the manual update. In Figure 13b the high dispersion of Young’s Modulus that are now spread in all parts of the belfry is represented. In fact, the lower values are now associated at different level, also in the internal vaults and not only on the bell cell.

In order to have a better look and determine the real state of the belfry, new experimental tests with a bigger array of sensors should then be performed, not only to reduce the dispersion on MAC but also to better capture the guessed distortion mode.

## 6. Conclusions

The research presented in this paper started from the direct observation of the dynamic characteristics of bell tower of Fermo’s Cathedral through OMA with the aim of comparing two different calibration procedures: one manual and the other automatic. In both procedures, only the stiffness matrices were perturbed, modifying the elastic modulus of the materials assigned in the NMs. With both methods the modal shapes of the NMs find correspondence with those of EM. In detail, by analyzing the results obtained with manual calibration, it was seen that there is a valid match between the numerical frequencies and the experimental ones since it is possible to have a greater control on the distribution of Young’s modulus. CrossMAC on the other hand did not give satisfactory results. With the automatic calibration based on a genetic algorithm the CrossMAC is much improved, while the frequencies have a higher deviation. The deviation between the frequency results can be explained by two different sets of things. The first being that the optimization with the genetic algorithm was done not only considering the frequency values, but also examining the CrossMAC, and the second being the possible influence of soil-structure interaction of the walls of the parts interfacing the bell tower which have not been directly instrumented and which, perhaps, could show a non-perfect interaction.

An easy modeling approach was adopted for the projection of the eigen-data between the approximated grid of EM and the refined mesh of NM, showing promise on the calibration of the CrossMAC between them. In future developments, the interpolations of the data on the nodes of the EM and their projection on the NM is going to be investigated to see if differences between simplified (coarser grid) and detailed models (fine grid) are present.

More detailed future studies may also concern the optimization of the position of the sensors within the structure in order to maximize and improve the information about the real dynamic parameters as much as possible. Further developments will concern the possibility of generating NMs with auto meshing, freeing itself from the need to bind to pre-set initial conditions which in any case limit the quality of the automation made.

## Figures and Tables

**Table 1:** Comparison between the Experimental Model (EM) and Numerical Model (NM) frequencies at step 0.

Mode	$f_{EM}\;[\mathrm{Hz}]$	Step 0 $f_{NM}\;[\mathrm{Hz}]$	$\Delta f_{0}\;(\%)$
1	1.654	1.337	19.16
2	1.800	1.671	7.17
3	4.236	3.876	8.49
4	4.955	4.182	15.60
5	9.767	7.404	24.19

**Table 2:** Comparison between EM and NM frequencies at step 1.

Mode	$f_{EM}\;[\mathrm{Hz}]$	Step 1 $f_{NM}\;[\mathrm{Hz}]$	$\Delta f_{1}\;(\%)$
1	1.654	1.500	9.31
2	1.800	1.903	−5.72
3	4.236	3.797	10.36
4	4.955	4.969	−0.28
5	9.767	8.516	12.81

**Table 3:** Comparison between EM and NM frequencies at step 2.

Mode	$f_{EM}\;[\mathrm{Hz}]$	Step 2 $f_{NM}\;[\mathrm{Hz}]$	$\Delta f_{2}\;(\%)$
1	1.654	1.610	2.66
2	1.800	1.871	−3.94
3	4.236	4.272	−0.85
4	4.955	5.036	−1.63
5	9.767	8.504	12.93

**Table 4:** Comparison between EM and NM frequencies at step 1 with Code Aster©.

Mode	$f_{EM}\;[\mathrm{Hz}]$	Step 1 $f_{NM}\;[\mathrm{Hz}]$	$\Delta f_{1}\;(\%)$
1	1.654	1.643	0.62
2	1.800	1.962	8.95
3	4.236	4.848	14.45
4	4.955	5.134	3.61
5	9.767	8.567	12.29

**Table 5:** Young’s Modulus values (E, MPa). Differences between the manual calibration and the Genetic Algorithms (GA) calibration for the step 1.

Number of Material	E [MPa] Manual Calibration	E [MPa] GA Calibration
1	2700	1000
2	3200	5090
3	2800	2230
4	2700	4540

**Table 6:** Comparison between EM and NM frequencies at step 2 with Code Aster©.

Mode	$f_{EM}\;[\mathrm{Hz}]$	Step 2 $f_{NM}\;[\mathrm{Hz}]$	$\Delta f_{2}\;(\%)$
1	1.654	1.529	6.97
2	1.800	2.000	1.95
3	4.236	3.715	23.36
4	4.955	5.095	0.76
5	9.767	9.686	13.05

**Table 7:** Young’s Modulus values (E, MPa). Differences between the manual calibration and the G.A. calibration for the step 2.

Number of Material	E [MPa] Manual Calibration	E [MPa] G.A. Calibration
1	1200	3797
2	2700	3425
3	1800	4005
4	1250	1554
5	1800	1283
6	1800	1592
7	4400	4473
8	3000	1258
9	2000	2917
10	3800	1092
11	2500	1970
12	1800	2322
13	800	4096
14	1500	3828
15	1200	4137
16	1800	3588
17	1800	2865
18	3200	3550
19	4400	3249
20	2800	2251
21	1900	1309
22	1800	3295
23	2400	871

## References

[1] Betti M., Vignoli A. (2011). Numerical assessment of the static and seismic behaviour of the basilica of Santa Maria all’Impruneta (Italy). Constr. Build. Mater..

[2] Milani G. (2013). Lesson learned after the Emilia-Romagna, Italy, 20–29 May 2012 earthquakes: A limit analysis insight on three masonry churches. Eng. Fail. Anal..

[3] Brandonisio G., Lucibello G., Mele E., De Luca A. (2013). Damage and performance evaluation of masonry churches in the 2009 L’Aquila earthquake. Eng. Fail. Anal..

[4] Acito M., Bocciarelli M., Chesi C., Milani G. (2014). Collapse of the clock tower in Finale Emilia after the May 2012 Emilia Romagna earthquake sequence: Numerical insight. Eng. Struct..

[5] Bartoli G., Betti M., Vignoli A. (2016). A numerical study on seismic risk assessment of historic masonry towers: A case study in San Gimignano. Bull. Earthq. Eng..

[6] Abruzzese D., Miccoli L., Yuan J. (2009). Mechanical behavior of leaning masonry Huzhu Pagoda. J. Cult. Herit..

[7] Preciado A. (2015). Seismic vulnerability and failure modes simulation of ancient masonry towers by validated virtual finite element models. Eng. Fail. Anal..

[8] Invernizzi S., Lacidogna G., Ramírez N.E.L., Carpinteri A. (2019). Structural monitoring and assessment of an ancient masonry tower. Eng. Fract. Mech..

[9] Clementi F., Gazzani V., Poiani M., Lenci S. (2016). Assessment of seismic behaviour of heritage masonry buildings using numerical modelling. J. Build. Eng..

[10] Lopez S., D’Amato M., Ramos L., Laterza M., Lourenço P.B. (2019). Simplified Formulations for Estimating the Main Frequencies of Ancient Masonry Churches. Front. Built Environ..

[11] Cabboi A., Gentile C., Saisi A. (2017). From continuous vibration monitoring to FEM-based damage assessment: Application on a stone-masonry tower. Constr. Build. Mater..

[12] Bartoli G., Betti M., Galano L., Zini G. (2019). Numerical insights on the seismic risk of confined masonry towers. Eng. Struct..

[13] Gentile C., Saisi A. (2007). Ambient vibration testing of historic masonry towers for structural identification and damage assessment. Constr. Build. Mater..

[14] Pierdicca A., Clementi F., Fortunati A., Lenci S. (2018). Tracking modal parameters evolution of a school building during retrofitting works. Bull. Earthq. Eng..

[15] García-Macías E., Ubertini F. (2019). Seismic interferometry for earthquake-induced damage identification in historic masonry towers. Mech. Syst. Signal Process..

[16] Balageas D., Fritzen C.-P., Gemes A. (2006). Structural Health Monitoring.

[17] Gentile C., Guidobaldi M., Saisi A. (2016). One-year dynamic monitoring of a historic tower: Damage detection under changing environment. Meccanica.

[18] Peeters B., Maeck J., De Roeck G. (2001). Vibration-based damage detection in civil engineering: Excitation sources and temperature effects. Smart Mater. Struct..

[19] Kita A., Cavalagli N., Ubertini F. (2019). Temperature effects on static and dynamic behavior of Consoli Palace in Gubbio, Italy. Mech. Syst. Signal Process..

[20] Dos Santos F.A., Cismasiu C., Cismasiu I., Fragiacomo M. (2018). Dynamic Characterisation and Finite Element Updating of a RC Stadium Grandstand. Buildings.

[21] Rainieri C., Fabbrocino G. (2014). Operational Modal Analysis of Civil Engineering Structures.

[22] Liu B., Ji Z., Wang T., Tang Z., Li G. (2018). Failure Identification of Dump Truck Suspension Based on an Average Correlation Stochastic Subspace Identification Algorithm. Appl. Sci..

[23] Lenci S., Consolini L., Clementi F. (2015). On the experimental determination of dynamical properties of laminated glass. Ann. Solid Struct. Mech..

[24] Benedettini F., Dilena M., Morassi A. (2015). Vibration analysis and structural identification of a curved multi-span viaduct. Mech. Syst. Signal Process..

[25] Pierdicca A., Clementi F., Isidori D., Concettoni E., Cristalli C., Lenci S. (2016). Numerical model upgrading of a historical masonry palace monitored with a wireless sensor network. Int. J. Mason. Res. Innov..

[26] Foti D., Diaferio M., Giannoccaro N.I., Mongelli M. (2012). Ambient vibration testing, dynamic identification and model updating of a historic tower. NDT Int..

[27] Bartz-Beielstein T., Branke J., Mehnen J., Mersmann O. (2014). Evolutionary Algorithms. Wiley Interdiscip. Rev. Data Min. Knowl. Discov..

[28] Escallón J., Wendeler C., Chatzi E., Bartelt P. (2014). Parameter identification of rockfall protection barrier components through an inverse formulation. Eng. Struct..

[29] Ferrari R., Froio D., Rizzi E., Gentile C., Chatzi E. (2019). Model updating of a historic concrete bridge by sensitivity- and global optimization-based Latin Hypercube Sampling. Eng. Struct..

[30] Leyder C., Chatzi E., Frangi A. (2017). Vibration-based model updating of a timber frame structure. Procedia Eng..

[31] Avendaño-Valencia L.D., Chatzi E. (2017). Sensitivity driven robust vibration-based damage diagnosis under uncertainty through hierarchical Bayes time-series representations. Procedia Eng..

[32] Qin S., Zhang Y., Zhou Y., Kang J. (2018). Dynamic Model Updating for Bridge Structures Using the Kriging Model and PSO Algorithm Ensemble with Higher Vibration Modes. Sensors.

[33] Pizzi A., Di Domenica A., Gallovič F., Luzi L., Puglia R. (2017). Fault Segmentation as Constraint to the Occurrence of the Main Shocks of the 2016 Central Italy Seismic Sequence. Tectonics.

[34] Ubertini F., Comanducci G., Cavalagli N., Pisello A.L., Materazzi A., Cotana F. (2017). Environmental effects on natural frequencies of the San Pietro bell tower in Perugia, Italy, and their removal for structural performance assessment. Mech. Syst. Signal Process..

[35] Rodrigues J. (2004). Identificação Modal Estocástica: Métodos de Análise e Aplicações em Estruturas de Engenharia Civil.

[36] Saisi A., Gentile C., Ruccolo A. (2017). Static and dynamic monitoring of a Cultural Heritage bell-tower in Monza, Italy. Procedia Eng..

[37] Peeters B., De Roeck G. (1999). Reference-based stochastic subspace identification for output-only modal analysis. Mech. Syst. Signal Process..

[38] Ministero delle Infrastrutture e dei Trasporti (2018). Aggiornamento delle ‘Norme Tecniche per le Costruzioni’—NTC: 2018 (in Italian).

[39] Ministero delle Infrastrutture e dei Trasporti (2019). Circolare 21 Gennaio 2019 n. 7 C.S.LL.PP. Istruzioni per l’applicazione dell’aggiornamento delle ‘Norme Tecniche per le Costruzioni’ di cui al D.M. 17/01/2018 (in Italian).

[40] Zhao Y., Du J., Bao H., Xu Q. (2018). Optimal Sensor Placement Based on Eigenvalues Analysis for Sensing Deformation of Wing Frame Using iFEM. Sensors.

[41] Slowik A., Kwasnicka H. (2020). Evolutionary algorithms and their applications to engineering problems. Neural Comput. Appl..

[42] Kokot S., Zembaty Z. (2009). Damage reconstruction of 3D frames using genetic algorithms with Levenberg–Marquardt local search. Soil Dyn. Earthq. Eng..

